# Network biology discovers pathogen contact points in host protein-protein interactomes

**DOI:** 10.1038/s41467-018-04632-8

**Published:** 2018-06-13

**Authors:** Hadia Ahmed, T. C. Howton, Yali Sun, Natascha Weinberger, Youssef Belkhadir, M. Shahid Mukhtar

**Affiliations:** 1 Department of Computer Science, University of Alabama at Birmingham, 115A Campbell Hall, 1300 University Boulevard, Birmingham, AL 35294 USA; 2 Department of Biology, University of Alabama at Birmingham, 464 Campbell Hall, 1300 University Boulevard, Birmingham, AL 35294 USA; 3 Gregor Mendel Institute (GMI), Austrian Academy of Sciences, Vienna Biocenter (VBC), Dr Bohr Gasse 3, 1030 Vienna, Austria; 4 Nutrition Obesity Research Center, University of Alabama at Birmingham, 1675 University Blvd, WEBB 568, Birmingham, AL 35294 USA

## Abstract

In all organisms, major biological processes are controlled by complex protein–protein interactions networks (interactomes), yet their structural complexity presents major analytical challenges. Here, we integrate a compendium of over 4300 phenotypes with Arabidopsis interactome (AI-1_MAIN_). We show that nodes with high connectivity and betweenness are enriched and depleted in conditional and essential phenotypes, respectively. Such nodes are located in the innermost layers of AI-1_MAIN_ and are preferential targets of pathogen effectors. We extend these network-centric analyses to Cell Surface Interactome (CSI^LRR^) and predict its 35 most influential nodes. To determine their biological relevance, we show that these proteins physically interact with pathogen effectors and modulate plant immunity. Overall, our findings contrast with centrality-lethality rule, discover fast information spreading nodes, and highlight the structural properties of pathogen targets in two different interactomes. Finally, this theoretical framework could possibly be applicable to other inter-species interactomes to reveal pathogen contact points.

## Abstract (web summary)

Nodes with high centrality in protein–protein interaction (PPI) networks are known to be essential in some organisms. Here, the authors in contrast find that in the interactome of *A. thaliana* central nodes are enriched in conditional and immune phenotypes and are preferred targets of pathogens.

## Introduction

**Fig. 1:**
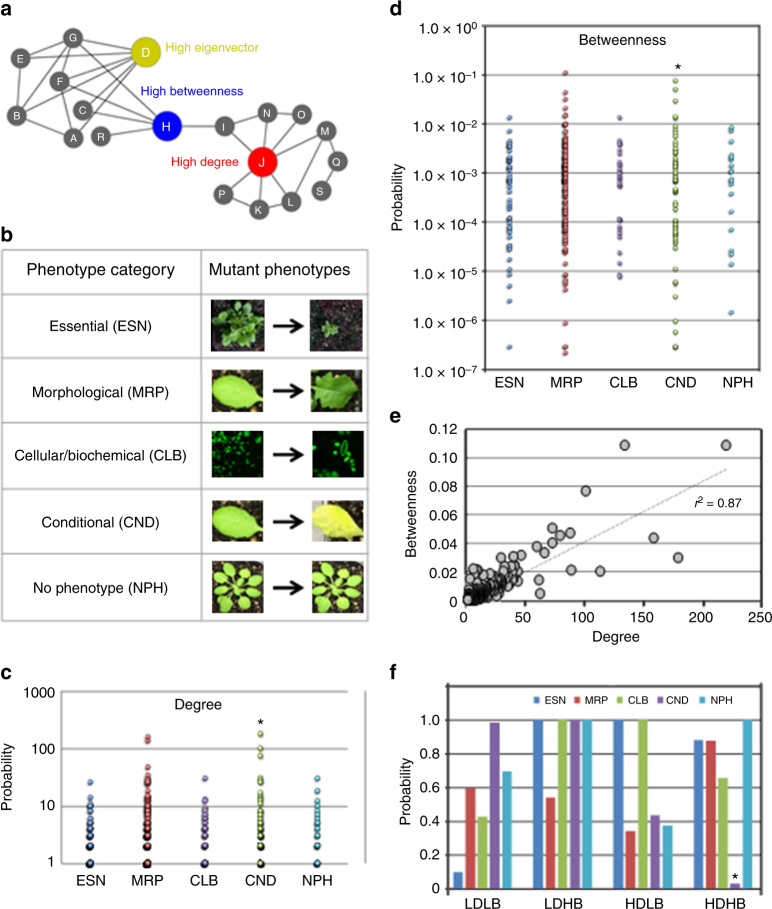
Hubs and bottlenecks are enriched in conditional phenotypes. **a** Schematic representation of high degree (hub; red), high betweenness (bottleneck; blue), and high eigenvector (green) nodes in a hypothetical network. **b** Cataloging loss-of-function mutant phenotypes in Arabidopsis based on five phenotypic groups: essential (ESN), morphological (MRP), cellular-biochemical (CLB), conditional (CND), and no phenotypes (NPH). **c**, **d** Node distribution corresponding to degree (**c**) and betweenness (**d**) for five phenotypic groups. CND phenotype enrichment for hub and betweenness are shown. **e** Relationship between node betweenness and degree distribution to identify high degree/high betweenness (HDHB), high degree/low betweenness (HDLB), low degree/high betweenness (LDHB) as well as low degree/low betweenness (LDLB) nodes (correlation coefficient of *r*^*2*^ = 0.87). **f** Hypergeometric test to determine the overrepresentation of a particular phenotypic group in nodes belonging to HDHB (*P* = 0.03), HDLB (*P* > 0.05), LDHB (*P* > 0.05), and LDLB (*P* > 0.05) categories

Networks consist of systems’ components, referred to as nodes and interactions between them, termed ‘edges’^1,2^. Network representation of a typical biological system constitutes the direct and indirect interactions among diverse molecular components. These molecular players, proteins in particular, participate in a wide range of biological processes, cellular pathways, and signaling cascades^1,3,4^. To achieve these cellular functions, proteins operate in conjunction with other partners, typically through direct physical protein–protein interactions (PPIs)^3,5^. The overall proteome-scale of these cellular interactions constitutes an “interactome”. Thus, elucidating the physical characteristics and functional interaction properties of an interactome could potentially reveal novel relationships between host proteins, new community structures as well as unique nodes with signaling cascades^6,7^. Such structural and functional topological features provide a range of information on individual nodes and edges, distinct modules, and the entire network as a whole^5,8,9^. Considering that diverse networks share similar organizational landscapes^10–12^, and the rate of information flowing through a network is dependent on the connectivity of its components^4^, several parameters of centrality measurements may act as indicators of important nodes in an interactome. For instance, network architectural properties can determine the connectivity and the critical distribution of a particular node within a network. These include degree, the number of connections of a node; betweenness, the fraction of the shortest paths that pass through a node; and eigenvector, a measure of the influence of a node in a network (Fig. 1a). Scale-free topology of a network follows a power law degree (a heavy-tailed) distribution exhibiting a few nodes with increased connectivity^1,4,8,13^. Recently, *k*-shell decomposition was shown to identify influential spreaders of information in social platforms and scientific publishing society^14^. Thus, deciphering the network architecture and understanding these topological properties could lead to the discovery of novel components in a complex system, which then provide biological insights as well as testable hypotheses.

Several proteome-scale interactomes have been generated in both prokaryotes and eukaryotes including human^6,15^, and the reference plant *Arabidopsis thaliana* (hereafter Arabidopsis)^7,16–18^. These not only mapped the network and module organization of protein interactions onto the overall cellular organization and function but also allowed understanding of genotype-to-phenotype relationships as well as evolution of biological networks and ancestral gene function^19^. As such, several studies in yeast interactomes suggest that high degree (hubs) and high betweenness (bottlenecks) are likely to be encoded by essential genes, a phenomenon termed as centrality-lethality rule^5,20–22^. In addition, PPI networks can also be exploited to decipher the complex interplay between hosts and their pathogens during the process of infection^3,4,23^. Analyses of inter-species interactomes demonstrated that proteins corresponding to hubs and bottlenecks are targets of pathogen attack^1,24,25^. Thus, a conceptual challenge posed by the centrality-lethality rule in analyzing inter-species interactome dataset stems from diverse lifestyles of pathogens on their hosts. Of particular interests are the pathogens that must keep their hosts alive (e.g. obligate biotrophs) throughout their life cycle. Therefore, association of hubs and/or bottlenecks (potential pathogens’ targets) with essentiality/lethality would principally undermine the pathogens’ infectious process. Thus, the phenotypic characteristics of nodes defined as hubs and/or other network centrality measures are a requisite layer of information to biologically understand inter-species interactome datasets.

Previously, we generated an Arabidopsis binary PPI map using ~8000 open reading frames representing ~30% of its protein-coding genes. Known as Arabidopsis Interactome version 1 “main screen” (AI-1_MAIN_), this network encompasses 5664 binary interactions between 2661 proteins^7^. We showed that AI-1_MAIN_ displays properties of a scale-free network that exhibits only 15 nodes with more than 50 interactions, i.e., ≥50 edges. These high-degree nodes are referred as hubs^50^. In addition, we also constructed two inter-species Plant–Pathogen Interaction Networks (PPIN-1 and PPIN-2)^26,27^ by systematically interrogating interactions between Arabidopsis proteins and pathogen proteins that are translocated inside the plant cells during infection (also termed pathogen effectors). Specifically, these effectors were derived from three distantly related pathogens^7,26,27^. Unexpectedly, however, we determined that these independently evolved effectors interact with a limited repertoire of 201 Arabidopsis proteins (hereafter host or effector targets). Subsequently, we demonstrated that these effectors can modulate host targets to establish effector-triggered susceptibility (ETS)^28–31^. We also showed that these targets participate in various layers of plant immunity including microbial-associated molecular patterns (MAMPs)- and Effector-Triggered Immunity (MTI and ETI, respectively)^32,33^. While most nodes corresponding to effector targets in AI-1_MAIN_ are highly connected (average degree), less than 6.5% of these nodes were defined experimentally as proteins belonging to the hub^50^ class. Thus, the predictive power of computational methods relying solely on centrality measures, particularly hubs, to determine if a given node in an interactome is more inclined to be targeted by pathogen effectors is limited^25^.

Here, we devise a method to predict effector targets in two unrelated experimental interactomes. To fully understand the functional interaction properties of the central nodes within a network, we curate a comprehensive dataset of ~4350 unique phenotypes in Arabidopsis. Unexpectedly, however, we demonstrate that hubs and bottlenecks are enriched in conditional phenotypes and depleted in essential phenotypes contrasting the centrality-lethality rule. We also discover that the nodes located in close proximity of the AI-1_MAIN_ core are targeted by effectors. We next apply this network topology framework to the extracellular LRR-based Cell Surface Interactome (CSI^LRR^), an unrelated experimental network that includes >500 interactions between membrane-localized leucine-rich repeat receptor kinases (LRR-RKs). Following centrality measure analyses, we predict a set of 35 LRR-RKs that are located near the core of CSI^LRR^ as the most influential nodes. Using two independent methods, we demonstrate that a subset of these predicted LRR-RKs can physically interact with bacterial effectors. Finally, we provide genetic evidence for the requirement of these newly discovered LRR-RKs modulating in plant immune system activities.

## Results

### Phenotypic properties of Arabidopsis hubs and bottlenecks

To examine the system-level relationship between genotype-to-phenotype in AI-1_MAIN_, we curated a comprehensive dataset of phenotypes corresponding to loss-of-function mutations in 4344 unique genes in Arabidopsis. We then categorized these genes into five functional groups: essential (ESN), morphological (MRP), cellular-biochemical (CLB), conditional (CND), and no phenotypes (NPH) as described by Lloyd and Meinke^34^ (Fig. 1b and Supplementary Data 1). Subsequently, we mapped these phenotypic groups onto the nodes of AI-1_MAIN_ and investigated their distribution in the network using enrichment assays for degree, betweenness, and eigenvector^7,26^ (Supplementary Fig. 1a, b). The definition of a high degree node (hub) in an interactome is arbitrary and perhaps depends upon the size and the density of a given network. For instance, we defined hubs with a degree greater than or equal to 50 (hub^50^) in the largest Arabidopsis interactome AI-1_MAIN_ as well as in PPIN-1 and PPIN-2^7,26,27^. However, the second largest Arabidopsis interactome, MIND1 (Arabidopsis Membrane-linked Interactome), described hub proteins with degree >70^17^. To demonstrate the robustness of our analysis, we implemented a second cut-off value for nodes displaying greater than or equal to 25 interactions (hub^25^). Given that high betweenness (bottlenecks) and high eigenvector cut-off values were not defined in either of the two largest Arabidopsis interactomes, AI-1_MAIN_ and MIND1, we also included two cut-off values each for high betweenness (bottleneck^0.025^ or bottleneck^0.01^) and high eigenvector (0.1 or 0.01) (Supplementary Data 2). Our analysis revealed that CND phenotypes are enriched in hub^50^ and hub^25^ (hypergeometric *P* < 0.05, Fig. 1c and Supplementary Data 2) as well as in bottleneck^0.01^ (hypergeometric *P* < 0.05, Fig. 1d and Supplementary Data 2) and bottleneck^0.025^ (hypergeometric *P* = 0.055, Supplementary Data 2). We also discovered that ESN phenotypes are enriched, although not statistically significant, in non-hubs (nodes with less than 25 edges) in AI-1_MAIN_ (hypergeometric *P* = 0.11, Supplementary Data 2). Finally, we did not observe a significant association of high eigenvector nodes in any of the above-mentioned phenotypes (Supplementary Fig. 2 and Supplementary Data 2). To control that the enrichment of CND phenotypes in hubs and bottlenecks is specific, we generated two random networks (“degree-preserving” and “non-degree-preserving”) encompassing nodes and edges similar to AI-1_MAIN_. Both random networks did not exhibit enrichment in any of the five phenotypes (Supplementary Fig. 3 and Supplementary Data 2). Thus, based on these analyses, we concluded that high degree (hubs) and high betweenness (bottlenecks) are enriched in CND but not in ESN phenotypes.

Enrichment of CND phenotypes with both hubs and bottlenecks prompted us to test whether high degree and high betweenness share significant fraction of the nodes with each other. Undoubtedly, we observed a strong positive correlation between degree and betweenness (Fig. 1e; *r*^2^ = 0.87). An analogous observation has been reported for Compound-Potential Target Network in cardiovascular disease^35^ (*r*^2^ = 0.77). However, the overlap of nodes corresponding to hubs or bottlenecks with high eigenvector did not yield any significant positive correlation (Supplementary Fig. 4; *r*^2^ = 0.55). Taken together, we showed that most central nodes in the network have a high degree and a high betweenness, and that most information perhaps flows through those important nodes. To analyze phenotypic groups’ enrichment assay on nodes that exhibit both hub and bottleneck properties, we categorized the nodes as high degree/high betweenness (HDHB), high degree/low betweenness (HDLB), low degree/high betweenness (LDHB), and low degree/low betweenness (LDLB). While HDHB nodes at two cut-off values were enriched in the CND phenotypic group (hypergeometric *P* < 0.05, Fig. 1f and Supplementary Data 2), no significant association of LDLB, HDLB, or LDHB nodes with any phenotypic functional groups was found. Finally, we did not observe enrichment of CND phenotypes with HDHB nodes in the two random networks (Supplementary Data 2). Thus, hubs and bottlenecks are enriched in CND phenotypes in AI-1_MAIN_, thereby contrasting the centrality-lethality rule. We also propose that Arabidopsis cells utilize hub and bottleneck proteins to regulate the flow and spread of information to a large number of proteins under diverse physiological conditions.

### Predictability of effector targets in plant interactome

Previous studies have shown that specialized pathogens have evolved sophisticated mechanisms to manipulate the key components of their hosts’ intracellular networks to their advantage^4,29^. Thus, we hypothesized that pathogens use effectors to target the most influential nodes in their host network. To test this concept, we determined if nodes corresponding to hubs, bottlenecks or high eigenvectors were more prone to be effector targets. Our results showed that high degree and high betweenness proteins (HDHB) are likely to be direct physical contact points of pathogen effectors, yet they only account for a small fraction of the range of effector targets determined experimentally in PPIN-1 and PPIN-2 (i.e. 6.45% and 18.71% for two cut-off values applied in our analyses, respectively) (Supplementary Fig. 5a and Supplementary Data 2). In addition, the target discovery rate of high eigenvector, HDLB, and LDHB with two cut-off values is lower than that of HDHB nodes (Supplementary Data 2). Given that PPIN-1 and PPIN-2 utilized effectors from three different pathogens, we also investigated whether a particular node targeted with more than one effector from the same pathogen or different pathogens could be used as a predictive indicator. However, we did not observe any correlation between the number of unique effectors interacting with a particular node and its degree in AI-1_MAIN_ (Supplementary Fig. 5b). In fact, the hub with the highest number of connections in AI-1_MAIN_ is targeted by only a single effector. Taken together, we concluded that centrality measures such as degree, betweenness, and eigenvector are thus of limited use to comprehensively analyze inter-species interactome datasets.

### Structural features of nodes in Arabidopsis interactome

**Fig. 2:**
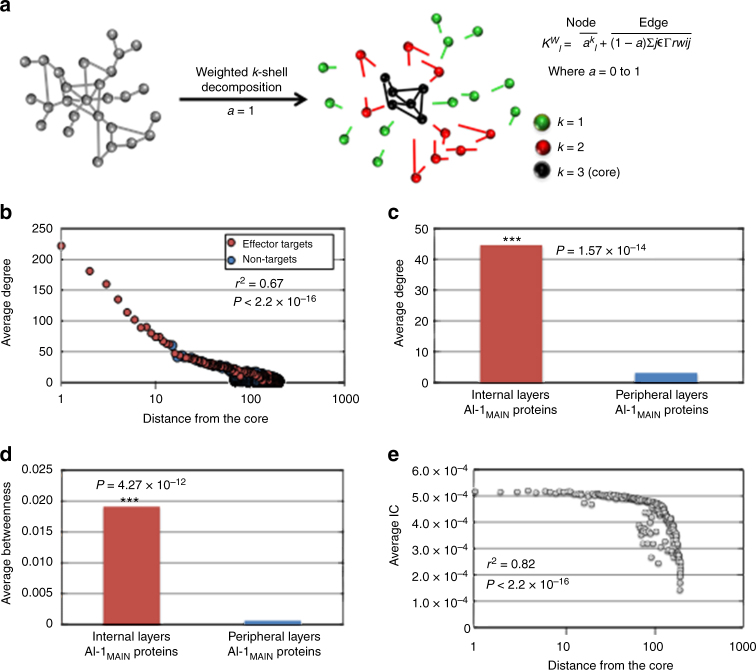
Network analyses of nodes in various layers of AI-1_MAIN_. **a** Schematic illustration of network layering using the weighted *k*-shell decomposition method. Connected hypothetical network (left; gray nodes) and decomposed network into three shells (right; *k* = 1, *k* = 2, and *k* = 3 in green, red, and black colors) are shown. **b** Distribution of average degree of each shell from the innermost of the network (core) designated as 1 to the periphery of the network denoted as 1000 in AI-1_MAIN_. Effector targets and non-targets are shown in red and blue nodes, respectively (*r*^2^ = 0.67 and Mann–Whitney–Wilcoxon Test *P* < 2.2 × 10^−16^). **c**, **d** Average degree (Welch’s *t*-test *P* = 1.57 × 10^−14^) (**c**) and average betweenness (Welch’s *t*-test *P* = 4.27 × 10^−12^) (**d**) for internal layers AI-1_MAIN_ proteins (red) and peripheral layers AI-1_MAIN_ proteins (blue) are plotted. **e** Distribution of average information centrality (IC) for each shell starting from the core of the network in AI-1_MAIN_ (*r*^2^ = 0.82 and Mann–Whitney–Wilcoxon test *P* < 2.2 × 10^−16^)

Recently, *k*-shell decomposition analysis was shown to outperform other known centrality measures including degree, betweenness, and PageRank in network-based analyses and for the identification of the most influential proteins in the network^14^. While the unweighted *k*-shell decomposition analysis considers all edges equally^36^, we used a weighted *k*-shell decomposition method to understand the topological properties of AI-1_MAIN_^37^ (Fig. 2a). We defined the internal and peripheral layers (shells) for AI-1_MAIN_ nodes that reside within the one-third and two-third layers, respectively (Supplementary Data 3). We observed a power-law correlation between the average degree and shell depth (*r*^2^ = 0.67 and Mann–Whitney-Wilcoxon Test *P* < 2.2 × 10^−16^) (Fig. 2b and Supplementary Fig. 6). We also demonstrated that the nodes located in the vicinity of the network core (internal layers AI-1_MAIN_ nodes) possess significantly higher average degree and betweenness in comparison to the nodes distributing in the periphery of the network (Fig. 2c, d, *P* = 1.57 × 10^−14^ and *P* = 4.27 × 10^−12^, respectively). These data indicate that the nodes residing within the internal layers are possibly better information spreaders. To substantiate this, we measured the information centrality (IC), an index that focuses on how information might flow through many different paths^13^. While we observed a strong power-law correlation between IC and shell depth (*r*^2^ = 0.82 and Mann–Whitney–Wilcoxon Test *P* < 2.2 × 10^−16^), we also showed that the average IC of nodes present in the internal layers of AI-1_MAIN_ is significantly higher than that of proteins in the remaining network (Fig. 2e and Supplementary Fig. 7, *P* < 2.2 × 10^−16^). These data indicate that the proteins closer to the network core are poised to be the most active spreaders of information.

### Effector targets in AI-1_MAIN_ by *k*-shell analysis targets

**Fig. 3:**
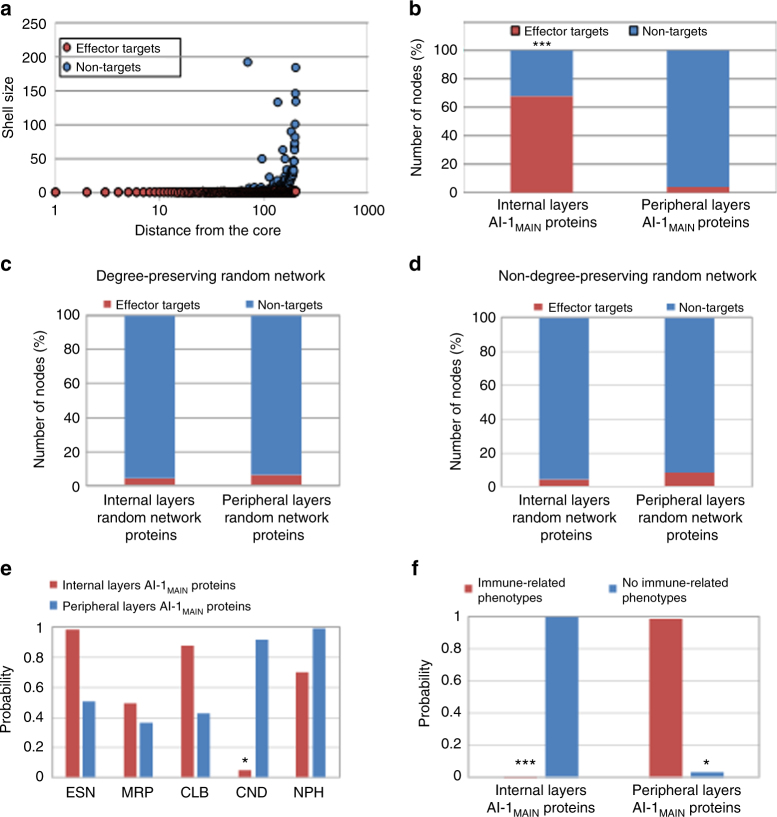
Functional properties of effector targets. **a** Distribution of effector targets (red) and non-targets (blue) within shells of AI-1_MAIN_ encompassing varied sizes as well as locations with reference to the core indicated as 1. A shell index ranges from 1 to 1000 in logarithmic scale is demonstrated. **b** Percentage of effector targets (red) and non-targets (blue) in two categories of nodes, internal layers AI-1_MAIN_ proteins and peripheral layers AI-1_MAIN_ proteins, are displayed (hypergeometric *P* = 2.61 × 10^−48^). **c**, **d** Distribution of effector targets (red) and non-targets (blue) within shells of degree-preserving random network (**c**) and non-degree-preserving random network (**d**) are shown. **e**, **f** Phenotypic overrepresentation analyses among the nodes of effector targets (red) and non-targets (blue). Enrichment of CND (hypergeometric *P* = 0.05) and immune-related phenotypes (hypergeometric *P* = 2.55 × 10^−6^) in **e** and **f**, respectively for effector targets (red) are shown. Overrepresentation of no immune-related phenotypes in nodes located in peripheral layers are demonstrated (hypergeometric *P* = 0.035)

Since the internal layers of AI-1_MAIN_ are enriched with nodes corresponding to influential spreaders of information, we thus predicted that effectors preferentially target nodes distributing in the vicinity of network core. Towards this, we demonstrated that nodes present in the internal layers of AI-1_MAIN_ are significantly enriched with effector targets compared to those located in the periphery of network (Fig. 3a, b, hypergeometric *P* = 2.61 × 10^−48^) with 33% discovery rate of effector targets (Supplementary Data 2, *P* = 3.01 × 10^−50^). No enrichment of effector targets was observed in randomly generated networks (Fig. 3c, d and Supplementary Data 2). In concordance with these results, we next showed that nodes that reside in the internal layers of AI-1_MAIN_ are enriched in CND phenotypes, and depleted in ESN phenotypes (hypergeometric *P* = 0.05, Fig. 3e and Supplementary Data 2) compared to the proteins in the periphery of the network. However, we did not observe any enrichment of these phenotypic groups in the internal layers of two independent random networks (Supplementary Fig. 8a and b and Supplementary Data 2). These results indicate that the weighted *k*-shell decomposition analysis surpasses other centrality measures for effector target discovery.

Previously, we performed a phenotypic mapping experiment of 124 Arabidopsis mutants corresponding to effector targets. In that study, we showed that 63 effector targets display disease-related phenotypes^27^, suggesting an almost equal chance (51%) to obtain immune-related phenotype or no phenotype for a mutant corresponding to an effector target. Remarkably, we demonstrated that the nodes located in the internal layers of AI-1_MAIN_ are enriched and depleted in immune-related phenotypes and no immune-related phenotypes, respectively (Fig. 3f and Supplementary Data 2, hypergeometric *P* = 2.552 × 10^−6^). This enrichment of immune-related phenotypes was absent in the internal layers of both random networks (Supplementary Fig. 9c and d and Supplementary Data 2). Intriguingly, we did not observe any correlation between the average effector degree (interacting degree of an effector to host proteins) and the proximity of the network core (Supplementary Fig. 9). Collectively, our data suggest that nodes located closer to the core of the network are targeted by effectors. Moreover, these nodes are enriched with CND and immune-related phenotypes.

### Discovery of the most influential nodes in CSI^LRR^

**Fig. 4:**
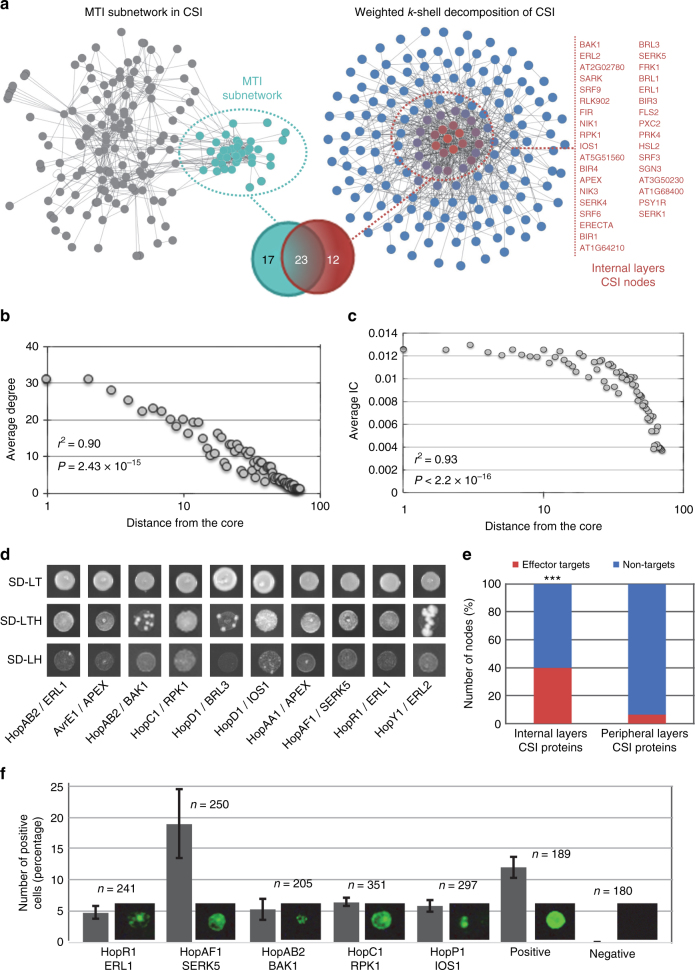
Experimental validation of the key proteins in CSI^LRR^. **a** CSI^LRR^ network is organized using Edge-weighted spring embedded layout (left) and weighted *k*-shell decomposition analysis (right). Internal layers of CSI^LRR^ proteins are annotated to the right (red). Venn diagram shows the overlap of 23 out of 35 nodes belonging to internal layers of CSI^LRR^ with MTI subnetwork. **b**, **c** Distribution of average degree (*r*^*2*^ = 0.9, Mann–Whitney–Wilcoxon test *P* = 2.43 × 10^−15^) (**b**) and average information centrality (IC; **c**) (*r*^2^ = 0.93, Mann–Whitney–Wilcoxon test *P* < 2.2 × 10^−16^) for each shell laid out from the core to the periphery of CSI^LRR^ network. **d** Pairwise yeast two-hybrid (Y2H) experiment between kinase domains of 20 LRR-RKs and 31 effectors from *Pseudomonas syringae* pv. tomato DC3000. An equal amount of mated diploid yeast is spotted on minimum synthetic medium dropouts SD-LT (leucine and tryptophan), SD-LTH (leucine, tryptophan, and histidine), and SD-LH (leucine and histidine + cycloheximide). SD-LTH ansd SD-LH media were supplemented with 1 mM 3-Amino-1,2,4-Triazol (3AT). Positive and negative interactions are determined based on growth and no growth on SD-LTH and SD-LH media, respectively. The identity of an LRR-RK and a particular effector for an interacting pair is revealed. **e** Phenotypic enrichment analyses among the nodes of effector targets (red) and non-targets (blue) among LRR-RKs belong to internal and peripheral layers CSI^LRR^ proteins are shown (hypergeometric *P* < 0.05). **f** Split-YFP interaction assay in protoplasts derived from wild-type leaves. The percentage of positive cells was calculated by dividing the number of fluorescing cells by the total number of cells within an image (indicated values = mean ± S.E.M.; six biological replications). *N* designates the number of cells evaluated. Representative photos of the positive interactions are shown. The CD3−1089::ADF4 (Arabidopsis Actin Depolymerizing Factor 4) and CD3−1096::MBP (maltose binding protein of *E. coli*) interaction was used as a positive control. Empty CD3−1089 and CD3−1096 vectors were used as a negative control in each independent transformation

LRR-RKs control plant growth and immunity by detecting and responding to ‘self’ and ‘non-self’ signals in the extracellular space. These surface localized receptors can act as pattern recognition receptors by sensing MAMPs, thereby controlling MTI^38–40^. Since a small subset of LRR-RKs have been shown to be targeted directly by pathogen effectors, we extended our weighted *k*-shell decomposition and functional analyses to identify both effector targets as well as the most influential spreaders of information in CSI^LRR^. Using our approach, we assigned 35 LRR-RKs to the one-third internal shells (or the internal layers) of CSI^LRR^ (Fig. 4a and Supplementary Data 3), and we postulated that these receptors are likely to be the most influential spreaders of information. Towards this, we performed additional network-centric analyses. We observed strong power-law correlations between the shell depth and the average degree (*r*^2^ = 0.9) or the average IC (*r*^2^ = 0.93) in CSI^LRR^ (Fig. 4b, c and Supplementary Fig. 10a, *P* = 2.43 × 10^−15^, *P* < 2.2 × 10^−16^, respectively). As in AI-1_MAIN_, the average degree value of the nodes located in the internal layers of CSI^LRR^ was significantly higher than that of their peripheral counterparts (Supplementary Fig. 10b*, P* < 0.001). Similar to AI-1_MAIN_^7^ and Compound-Potential Target Network in cardiovascular disease^35^, we discovered a significant overlap of nodes between high degree and high betweenness in CSI^LRR^ network (Supplementary Fig. 10c, *r*^2^ = 0.71). Thus, although generated by independent methods, CSI^LRR^ and AI-1_MAIN_ share an overall similar network architecture based on centrality measures and weighted *k*-shell decomposition analyses.

Based on the network topological similarity concept between CSI^LRR^ and AI-1_MAIN_, we predicted that the nodes present in the internal layers of CSI^LRR^ should be associated with CND and immune-related phenotypes including MTI. Our analysis was limited by the dearth of LRR-RKs for which a clearly defined function has been assigned in the literature. However, we found that BRI1-associated receptor kinase 1 (BAK1), the most interconnected node in CSI^LRR^ ^16^, is located in the core of CSI^LRR^. BAK1 acts as a major coreceptor for a range of ligand binding receptors that regulate MTI and plant development, and is, therefore, also a functional hub^38^. It is worth noting that none of these LRR-RKs have been previously associated with ESN phenotypes, further suggesting the roles of this set of LRR-RKs in stress responses.

In addition to the roles of BAK1^38^, the functions of 22 other LRR-RKs including somatic embryogenesis receptor kinases (SERKs)^38^, BAK1-interacting LRR-RKs (BIRs)^39^, Brassinosteroid insensitive 1 (BRI1)-LIKE (BRLs)^41^, ERECTA (ER)^40^, ER-like (ERL1s)^40^, flg22-induced receptor-like kinase 1 (FRK1)^29,42^, Impaired Oomycete Susceptibility 1 (IOS1)^43^, Receptor Protein Kinase 1 (RPK1)^44^, Senescence-Associated Receptor-Like Kinase (SARK)^45^, Articulation Point Executive (APEX)^16^, Flagellin Sensitive 2 (FLS2)^46^, HAESA Like (HSL2)^47^, Strubbelig Receptor Family 3 (SRF3)^48^, and PSY1-receptor (PSY1R)^49^ have been previously proposed in MTI as well as other biotic and abiotic stresses (CND phenotypes). To further substantiate the potential functions of these 35 LRR-RKs in CND as well as immune-related phenotypes, we compared them with an MTI subnetwork^16^. This immune-related module was derived through a community analysis in CSI^LRR^. We found that LRR-RKs located in the internal layers of CSI^LRR^ constitute 66% of the MTI subnetwork (Fig. 4a).

Given the overwhelming enrichment of LRR-RKs corresponding to CSI^LRR^ internal layers with CND and immune-related phenotypes, we further hypothesized that these sets of LRR-RKs are potential targets of pathogen effectors. To test this, we performed a pairwise Yeast two-hybrid (Y2H) experiment and tested cytoplasmic domains of 20 LRR-RKs against 31 effectors from *Pseudomonas syringae* pv. tomato DC3000 (*Pto* DC3000). We recapitulated the interaction of BAK1 with HopAB2, originally discovered in split-ubiquitin system^46^. Moreover, we also found seven additional LRR-RKs interacting with nine effectors (Fig. 4d, e; 40% effector discovery rate, *P* < 2.2 × 10^−16^). In contrast, a parallel experiment involving LRR-RKs that distribute in the peripheral layers of CSI^LRR^ showed no significant enrichment of effector target discovery rate (6.25%; Fig. 4e). We further validated these inter-species interactions by employing split-YFP system in Arabidopsis cells, an independent confirmatory method (Fig. 4f). Thus, we expected the internal layers CSI^LRR^ LRR-RKs to be the converging points of effectors from diverse pathogens. Indeed, FLS2 was previously demonstrated to associate with a bacterial effector, AvrPto in a co-immunoprecipitation assay^50^. Moreover, three NSP-Interacting Kinases, NIK1, NIK2, and NIK3, were previously shown as virulence targets of the geminivirus nuclear shuttle protein (NSP)^51^, further substantiating the discovery rate of effector targets located within the internal layers of CSI^LRR^.

### Immune-related functions of newly identified LRR-RKs

**Fig. 5:**
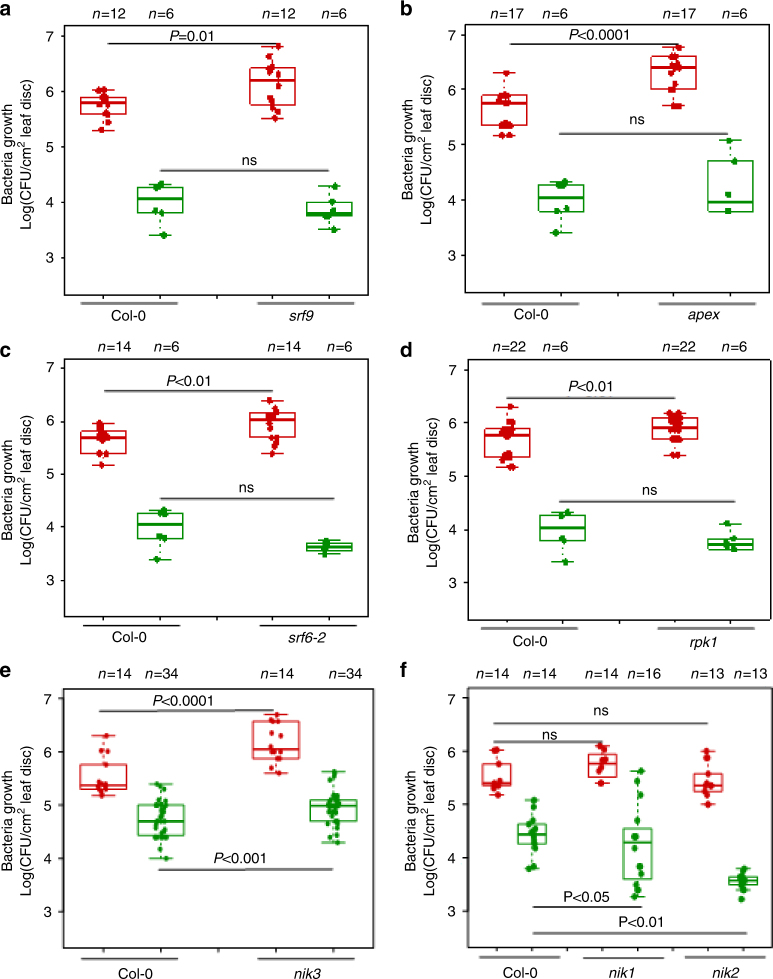
Immune-related functions of novel LRR-RKs in CSI^LRR^. Bacterial growth of *Pseudomonas syringae* pv. *tomato* DC3000 (*Pto* DC3000, red bars) and effectorless mutant strain *Pto* DC3000 hrcC− (green bars) were quantified 3 days after syringe inoculation (OD_600nm_ = 0.0002) on *srf9* (**a**), *apex* (**b**), *srf6-2* (**c**), *rpk1* (**d**), *nik3* (**e**), and *nik1* as well as *nik2* (**f**). Wild-type Col-0 plants were used as controls. Each dot in the box and whisker plot represents individual data points. *n* shows the number of leaf samples, and each sample contains four biological independent leaf discs. One-way ANOVA was performed to estimate statistical significance for bacteria growth. n.s. stands for not significant. *P < 0.05, **P < 0.01 and ***P < 0.001

In addition to the known CND and immune phenotypes for 22 LRR-RKs, we aimed to characterize the roles of seven additional LRR-RKs in plant immunity (MTI and ETS). We obtained loss-of-function mutants corresponding to NIK1, NIK2, NIK3, SRF6, SRF9, RPK1, and APEX and demonstrated the lack of transcript accumulation in these mutants^16^ (Supplementary Fig. 11). We hypothesized that mutants corresponding to these seven LRR-RKs would manifest CND and immune-related phenotypes. To test this, we subjected the mutants corresponding to these seven LRR-RKs to infection with either the fully virulent bacterial pathogen *Pto* DC3000 or with *Pto* DC3000 hrcC−, a mutant strain that lacks a functional type-III secretion system required for effector protein delivery into host cells. While we reproducibly observed a significant increase in the virulence of *Pto* DC3000 on the *srf9*, *apex*, *srf6-2*, *rpk1*, and *nik3* mutants compared with wild-type Col-0 plants (Fig. 5), no significant difference in bacterial growth was observed when plants were infected with *Pto* DC3000 hrcC− except for *nik3* (Fig. 5). These results indicate that SRF9, APEX, SRF6, and RPK1 LRR-RK receptors have an MTI-independent function and negatively regulate the virulence activities of one or more effectors. In comparison to wild-type plants, we observed a significant reduction of *Pto* DC3000 hrcC− growth in the *nik1* and *nik2* mutants, whereas *Pto* DC3000 growth was unaffected (Fig. 5f). Thus, NIK1 and NIK2 negatively regulate the induction of MTI. Overall, we demonstrated the positive and negative contributions of these seven LRR-RKs in MTI as well as ETS under diverse physiological conditions. While the molecular mechanisms by which these newly identified LRR-RKs contribute to plant defense are focal points of future research, here we discovered novel players of plant immunity in CSI^LRR^ using network biology-based approaches.

## Discussion

In the last 15 years, interactome mapping in diverse organisms led to the development of several premises in network biology^4,5,15,25^. Scale-free network architecture, nodes’ connectivity, and the centrality-lethality rule are applied to discover novel components in diverse systems. In this study, we performed an in-depth network analyses on two unrelated experimental interactomes and revealed their topological features. We determined that high degree and high betweenness nodes are enriched and depleted in conditional and essential phenotypes, respectively. Additional noteworthy findings concern another widely known network model implying that highly connected and central nodes are targets of diverse pathogens. Instead, we demonstrated that nodes with increased connectivity that are located closer to the network core are the preferred targets of pathogen attack compared to the proteins that reside in the network periphery. Finally, we identified previously known as well as novel LRR-RKs involved in MTI and ETS.

We showed that both AI-1_MAIN_ and CSI^LRR^ displayed properties of a scale-free network^7^ (Fig. 4 and Supplementary Fig. 1). Since the birth of this theory, however, several seminal studies have outlined sentinel importance^10–12^ or presented contradicting views of the scale-free property^52–55^. An important question, however, is whether the power law distribution of nodes is a consequence of a specific technology bias, for example, yeast two-hybrid (Y2H)^56^ vs. affinity purification with mass spectrometry (AP-MS)^57^. Irrespective to the choice of research methods, dozens of large-scale interactomes in both prokaryotes and eukaryotes have been reported to exhibit scale-free properties^4,5,15,25^. In Arabidopsis, AI-1_MAIN_ was generated using GAL4-based Y2H method by employing over 8000 ORFs^7^. While a systems-level approach was used, it is still arguable that cloning bias to the short fragment ORFs and network incompleteness could have contributed to scale-free network topology. It is worth noting, however, that a family-wide collection of LRR-RKs clones was used to generate CSI^LRR^ by applying a fundamentally different proteome technology^16^. Similarly, the Arabidopsis Membrane-linked Interactome (MIND1) was constructed by employing split-ubiquitin system and a comprehensive list of over 3200 signaling and membrane-bound proteins^17^. Both CSI^LRR^ (*r*^2^ = 0.82) and MIND1^17^ display properties of scale-freeness with a similar confidence value compared to AI-1_MAIN_ (*r*^2^ = 0.86). Thus, methodological biases, if any, in these Arabidopsis interactomes have no influence on the scale-free property of network architecture.

Our findings unequivocally demonstrate that essential phenotypes are depleted in nodes corresponding to hubs and bottlenecks in contrast to the concept of the centrality-lethality rule (Figs. 1, 4, 5). According to this important premise of network property, disabling highly connected nodes or hubs may entirely dismantle the network. Having provided for the initial discovery of hubs as essential nodes in the yeast interactome, this network principle was further expanded on bottlenecks as well as interactomes in both prokaryotes and eukaryotes^5,20–22^. In addition, essentiality was also investigated on the size of the complexes^58^ as well as different kinds of hubs including party and date hubs or single- and multi-interfaced hubs^59^. However, controversy surrounded this topic as soon as additional proteome-scale interactomes were generated in yeast, fly, worm and human^24,59,60^. In these unrelated studies, network analyses did not show any positive correlation between degree and essentiality. Our discovery indicating depletion of an essential category of phenotypes in hubs and bottlenecks agrees with another study performed in yeast and worm pertinent to network connectivity and evolution. Kafri et al. (2008)^61^ showed that hubs are more frequently associated with functionally redundant gene duplicates. It was also suggested that this functional redundancy perhaps buffers against mutations, and thus minimizes the lethality impact of these “so called” vulnerable nodes^61^. Regardless of these discrepancies, hubs and bottlenecks remained important with respect to inter-species interactions such as host-microbe interactomes (discussed below). While previous network analyses were performed to investigate a correlation between essentiality and hubs as well as bottlenecks, a question that remains to be addressed is in what types of phenotypes are these highly connected and central nodes enriched? By utilizing a compendium of over 4300 phenotypes (Fig. 1), we showed that hubs and bottlenecks are enriched in conditional phenotypes. Thus, our data take a step towards highlighting the importance of these highly connected and central nodes. Moreover, this discovery will pave the path for future studies in conjunction with biotic and abiotic stresses in plants and other eukaryotes.

The next question we addressed in this study was whether diverse centrality measures can be used as the predictors of pathogen attack (Figs. 1 and 2). Previously, network topology analyses revealed that nodes encoding hubs and bottlenecks are targeted by pathogen virulence factors as well as associated with oncogenesis and other human diseases^3,6,25,62–66^. Similar to these findings, we previously demonstrated that almost all of the hub^50^ nodes in AI-1_MAIN_ are targeted by effectors from diverse pathogens. While hubs and bottlenecks are remarkable predictors of pathogen targets, they only make up for a small fraction of nodes in a scale-free network, i.e., 6.5% in AI-1_MAIN_. These data indicate that hubs and bottlenecks can predict pathogen effectors in Arabidopsis with high significance as shown for human–viral or human–bacterial interactomes^24,25,64,67^, but the predictive power of these centrality measures is very low. Given that infectious organisms require the hosts to remain viable for their growth and reproduction, a very recent report suggests that interactome connectivity directly relates to pathogen fitness during infection^24^. According to this tenant, pathogens rearrange host interactomes instead of dismantling network integrity to alter cellular physiology for their benefits^24^. Thus, we expected that pathogens rewire their host’s interactome by interfering with the most influential nodes. In our study, therefore, we expanded our network centrality measures analyses to weighted *k-*shell decomposition and determined the best information spreaders in AI-1_MAIN_ and CSI^LRR^ (Figs. 2, 4). Indeed, it was previously shown that *k*-shell outperforms widely used centrality measures in diverse social networks. Likewise, our *k*-shell analysis discovered the occurrence of 33% of effector targets compared to a small fraction of hubs/bottlenecks in AI-1_MAIN_. We also showed that majority of the effector targets are located near to the interactome core rather than periphery of the network. These nodes in the vicinity of core exhibit increased average degree, betweenness, and IC as well as enriched in immune-related and conditional phenotypes. Remarkably, a recent report demonstrated that the best information spreaders are located in the *k*-cores of a wide range of networks including Twitter, Facebook, LiveJournal, and scientific publishing society^14^. These results from social networks further validate our data in a biological interactome context.

Another novel discovery of our study is the experimental validation of our predictions in an unrelated experimental network, CSI^LRR^ (Fig. 5). LRR-RKs have been implicated in diverse physiological programs including developmental processes and plant immune systems, in particular MTI^38–40^. These cell surface receptors bind with extracellular signaling molecules, transduce the information through a downstream signaling cascade, and activate a fine-tuned cellular response. Generally, this is accomplished by dynamic association and dissociation of receptor–coreceptor complexes as well as integration of synergistic and antagonistic signaling outputs triggered by diverse LRR-RKs^38–40^. Among the 35 most influential nodes discovered using this analytical framework, we found BAK1, a functional hub of plant immunity and developmental processes, in the core of CSI^LRR^. In addition, none of these internal layer nodes exhibited essential phenotypes but rather immune-related and conditional phenotypes, further suggesting the specificity and wider applications of our analysis. While our experimental approach unveiled new players in MTI and ETS, there are several open questions that may form the basis for future studies. For instance, how these LRR-RKs exert synergistic and antagonistic actions to transduce fine-tuned immune, growth and developmental signals and how pathogen effectors mechanistically interfere with this balanced defense responses? Another question concerns the dynamicity of the complexes involving LRR-RKs under diverse physiological conditions. Likewise, an area of research that needs to be explored is whether and if so, how pathogens’ apoplastic effectors and other molecules target extracellular interface of these LRR-RKs. Finally, it remains to be determined whether effectors from diverged pathogens also target LRR-RKs from internal layers of CSI^LRR^.

**Fig. 6:**
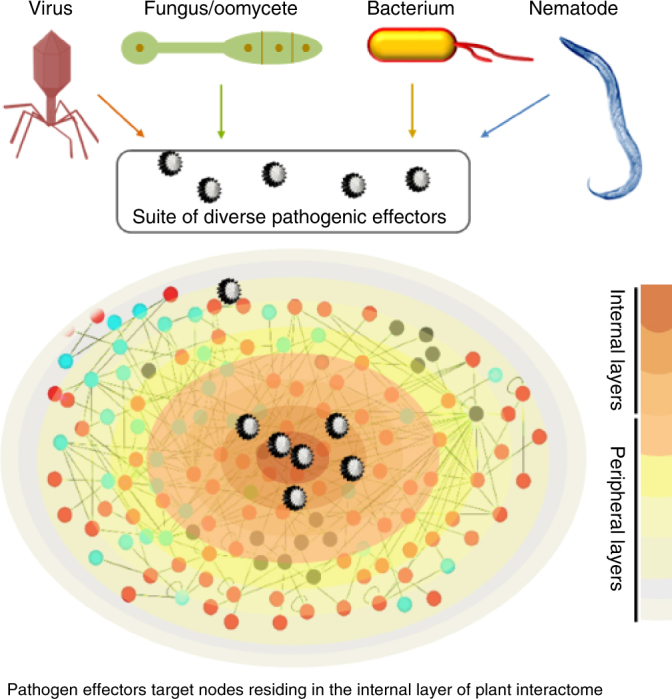
A model illustrating effector targets in plant interactome. Plant protein–protein interaction network (interactome) exhibiting direct physical interactions is demonstrated in the internal and the peripheral layers. Viral, fungal/oomycete, bacterial, and nematode pathogens delivering suite of pathogenic effectors are shown. A key to the color scheme representing the internal and the peripheral layers is revealed

Taken together, we convincingly demonstrated that connectivity itself, but not hubs and bottlenecks per se, are the indicators of pathogen virulence targets. We also determined that network decomposition analysis, in conjunction with connectivity, would allow researchers to identify most influential and vulnerable points in the network (Fig. 6). This work elucidates the topological and functional properties of effector targets, while successfully predicting the most influential spreaders of information and experimentally determining nodes that are exceptionally vulnerable to pathogen attack. The detailed curation of our Arabidopsis phenotypic dataset can be useful to the scientific community for additional genome-to-phenome studies. Our network-centric approach has exciting potential applicability on diverse intra- and inter-species interactomes including human PPI networks in efforts to unravel host–pathogen contact points, while fostering the design of targeted therapeutic strategies.

## Methods

### Network analyses

The centrality measures in both Arabidopsis Interactome version 1 “main screen” (AI-1_MAIN_) and Cell Surface Interactome (CSI^LRR^) were analyzed using Networkx package and Python 2.7.10. Briefly, we calculated degree, the *n* number of edges of a particular node, and degree distribution of a network is defined as *n*_*k*_*/n*. Betweenness, the number of shortest paths that pass through a node (*v*), is analyzed as

$$g\left( v \right) = \mathop {\sum }\limits_{s \ne v \ne t} \frac{{\sigma _{st}(v)}}{{\sigma _{st}}},$$

where *σ*_*st*_ is the sum of shortest paths from node “*s*” to node “*t*”; *t* and *σ*_*st*_(*v*) is the number of paths that pass through (*v*). Eigenvector, a measure of the influence of a node in a network, *x*_*i*_ of node i, is calculated as *x*_*i*_=1/λ∑_*k*_*a*_*k*,*i*_*x*_*k*._ For each node we computed its degree, betweenness, and eigenvector as described above. Hence, we selected two cut-offs for each case.

Degree: 50 and 25

Betweenness: 0.025 and 0.01

Eigenvector: 0.1 and 0.01

IC calculates the flow of information between two nodes in a connected network. IC was computed as described previously^13^. Briefly, IC (*i*) for node *i* in a graph G is calculated as

$${\mathrm {IC}}\left( i \right) = \left[ {\frac{1}{n}\mathop {\sum }\limits_j \frac{1}{{I_{ij}}}} \right]^{ - 1}.$$

Here, *n* is the total count of nodes and *I*_*ij*_=(*r*_*ii*_+*r*_*jj*_−*r*_*ij*_)−1, *r*_*ij*_ is a component of *R* matrix. *D* is a weighted degree diagonal matrix for each node, and *J* is a matrix consisting 1 for all elements. Therefore, *R*=(*r*_*ij*_)=[*D*−*A*+*J*]−1. Mathematically, *I*_*ii*_ is well-defined as infinite. Hence, ${\textstyle{1 \over {I_{ij}}}} = 0.$

Weighted *k*-shell decomposition is performed as described in Fig. 2 and Wei et al.^37^. Briefly, the generation of shells process is defined by the weight of both degree of a node and its edges and calculated as ${{k}}_{{i}}^{{W}} = {{\alpha k}}_{{i}} + \left( {1 - {{\alpha }}} \right)\mathop {\sum}\nolimits_{{{j}} \in \Gamma {{i}}} {{{W}}_{{{ij}}}}$, where Γ_*i*_ are a set of neighboring nodes of *i*. *w*_*ij*_ is the weighted of the edge that is defined as *w*_*ij*_ = *K*_*i*_ + *K*_*j*_. The value of *α* can be set on a spectrum of 0 through 1 with 0 and 1 determining high edge or high degree favorability in *k*-shell decomposition calculation, respectively. We performed *k*-shell decomposition using a range of *α* cut-off values, i.e., 0, 0.5, and 1.0.

While this method has been previously described, the code for weighted *k*-shell decomposition was not available. We implemented the weighted *k*-shell algorithm in Java language and can be accessed at goo.gl/c5ISSe. Average degree was calculated by summing the degree of each node in the shell and dividing by the number of nodes presented in the shell using the following formula Shell_avgDegree_*i*_ = Σ_*j*∈*S*_ *k*_*j*_/*N*. Where *S* is the set of nodes in the shell_*i*_ and *N* is the number of nodes in the shell_*i*_. Similarly, average betweenness and average IC for each shell were analyzed. CSI^LRR^ network is visualized using Cytoscape 3.4^68^.

### Statistical analyses

We calculated hypergeometric test, linear regression (*r*^2^), Mann–Whitney–Wilcoxon test, and Welch’s *t*-test using R version 3.3.1 as well as online Stat Trek tool. Briefly, hypergeometric test was performed to determine the enrichment of five phenotypic groups: (1) (essential (ESN), (2) morphological (MRP), (3) cellular-biochemical (CLB), (4) conditional (CND), and (5) no phenotypes (NPH), two immune phenotype classes: (1) immune-related phenotypes and (2) no immune-related phenotypes as well as frequency of targets among nodes with diverse centrality measures. The following centrality measures were utilized: degree, betweenness, eigenvector, high degree/high betweenness (HDHB), high degree/low betweenness (HDLB), low degree/high betweenness (LDHB), and low degree/low betweenness (LDLB) with two different cut-off values, internal and peripheral layers AI-1_MAIN_ proteins. Linear regression and Mann–Whitney–Wilcoxon test were performed on average degree and distance from the core, average IC and distance from the core for both AI-1_MAIN_ and CSI^LRR^. Welch’s *t*-test was performed to compare the degree and betweenness of internal and peripheral layers AI-1_MAIN_ proteins in the network.

### Arabidopsis loss-of-function phenotypes database

We generated a database of 4344 unique Arabidopsis genes with loss-of-function mutant phenotypes. Briefly, we categorized genes in five prioritized phenotypic groups: essential (ESN), morphological (MRP), cellular-biochemical (CLB), conditional (CND), and no phenotypes (NPH) as described by Lloyd and Meinke^34^. Two thousand four hundred genes with loss-of-function mutant phenotypes were included from Lloyd and Meinke^34^. In addition, we downloaded genome-wide phenotypes from TAIR10^69^ and curated additional 1944 phenotypes making a comprehensive database of 4344 Arabidopsis genes with mutant phenotypes.

### Plant cultivation and mutants

The wild-type used in all experiments was Arabidopsis accession Columbia (Col-0). The following mutant plant genotypes were used in this work: *nik1* (SALK_017538C), *nik2* (SALK_044363C), *nik3* (SALK_092902), *srf6-1* (SALK_054337C), *srf6-2* (SALK_077702), *srf9* (SALK_011495C), *rpk1*(SALK_005054), *apex* (SALK055240), and *efr fls2*^43^. The insertion sites for the T-DNA lines were located in the open reading frames of the genes and were genotyped by PCR prior to use.

### Characterization of mutant lines

T-DNA lines were genotyped by PCR using DNA extracted from leaf tissue of mutant lines with Col-0 used as a control. Presence of a T-DNA insertion was confirmed with a primer combination of LBb1.3 with an RP primer specific for each T-DNA insertion line. An intact WT locus was tested with an LP and RP primer combination. Used primer sequences shown in Table 1, LBb1.3 (5′-ATTTTGCCGATTTCGGAAC-3′) was used as left border primer for T-DNA insertion for all genotypes.

**Table 1:** List of the primers used in this study

Gene	Name of primer	Sequence 5′–3′
*NIK1*	SALK_017538-LP	GACAAAAACATGACAGGGTGG
	SALK_017538-RP	CATTGTTTTCCTTGCTTGCTC
*NIK2*	SALK_044363-LP	CCAAAGAAGAAAACCAAAGCC
	SALK_044363-RP	AGAGAAGCTCCAAGCCAAAAC
*NIK3*	SALK_092902-LP	ATTACACCTTTCTGGTGTGCG
	SALK_092902-RP	TGAAGGGTAATTGGTAATGCG
*SRF6-1*	Salk_054337_LP	AACGACTTTCACGGTATGCAC
	Salk_054337_RP	TGTCAAATGGTTTTCTCCCAG
*SRF6-2*	Salk_077702_LP	TCGAGTTTATAACCGTCGGTG
	Salk_077702_RP	TGTGTGCATACCGTGAAAGTC
*SRF9*	SALK_011495-LP	TCCCTGTCCACTCAAACAAAG
	SALK_011495-RP	ATCCCTGTTTCACCCTTCTTC
*RPK1*	SALK_005054_LP	CTCATGTCACAACTGGTGTGG
	SALK_005054_RP	ACAAAGCCAACAAATCGTTTG
*APEX*	SALK_055240_LP	GCATAAGCCATTTTCCCAAAC
	SALK_055240_RP	TCATGGAAACTTTCACCGTTC

### Quantification of mRNA with qPCR

For transcript levels accumulations in the mutants corresponding to LRR-RKs, RNA was extracted from leaf tissue using a GeneMATRIX Universal RNA Purification Kit (EURX). cDNA was synthesized using High Capacity cDNA Reverse Transcription Kit (Applied Biosystems). qPCR assays were performed with FastStart Essential DNA Green Master (Roche) using 2.5 µl of diluted cDNA in 10 µl total reaction. Specific primers were used at a final concentration of 1 µM with following sequences: Actin 2/8 Fw qPCR (5′-TCTTGTTCCAGCCCTCGTTT-3′), Actin 2/8 Rv qPCR (5′-TCTCGTGGATTCCAGCAGCT-3′) for normalization of gene of interest.

Real-time qPCR was operated with a Roche LightCycler96 and data analyzed using the accompanying LightCycler96 Version 1.1 software. Relative gene expression levels were calculated using the 2^−ΔΔ*C*^_*T*_ method.

### Pathogen assays and chemicals

Pathogen infection was performed as described in^13^. Briefly, four-week-old plants were syringe-infiltrated with *Pseudomonas syringae* pv. *tomato* DC3000 (*Pto* DC3000) or an effectorless mutant strain, *Pto* DC3000 hrcC− with bacterial solution OD600 nm = 0.0002 in 10 mM MgCl_2_. Four leaves per plant and five/six plants per genotype were used for pathogen quantification through serial dilution. Four independent biology replicates were combined for data analysis.

### Yeast two hybrid

The yeast two-hybrid (Y2H) experiment was performed similar to Mukhtar et al. with some adjustments^7,26,56^. We used 20 of the 35 leucine-rich repeat receptor protein kinases (LRR-RKs) that we predicted by our network analysis to be effector targets and 31 effectors from *Pseudomonas syringae* pv. *tomato* DC3000 in both bait and prey plasmids. The bait proteins were fused to the DNA binding domain of GAL4 using a pDEST-DB vector with a leucine selection marker, while the prey proteins were fused with the GAL4 activation domain in a pDEST-AD-CYH vector with a tryptophan selection marker. Each interaction was tested in both directions. Prey and bait plasmids were transformed into haploid *Saccharomyces cerevisiae* strains Y8800 (MATa) and Y8930 (MATα), respectively, and confirmed by selecting on their corresponding selective media.

Haploid bait and prey strains were mated in liquid YEPD (yeast extract 10 g/L, peptone 20 g/L, dextrose 20 g/L, adenine 100 mg/L) media overnight at 30 °C. The resulting cultures were transferred to SD-LT media for 48 h in order to select for diploid yeast. The reconstitution of GAL4 transcription factor through the interaction of the bait and prey leads to the initiation of a *HIS3* reporter gene and consequently biosynthesis of histidine. Since the pDEST-AD vector contains the *CYH2* (*a cycloheximide sensitive gene*), any growth on the yeast media containing cycloheximide constitutes a false-positive interaction. Equal amounts of diploid yeasts were transferred to solid SD-LTH (positive selection plates) and SD-LH + cycloheximide (20 mg/L) media (de novo autoactivation plates). Additionally, the histidine biosynthesis inhibitor, 3-amino-1,2,4-triazole (3-AT), was added to solid media to increase the stringency of the experiment and reduce any background^7,26,56^. Positive interactions were scored owing to yeast growth on positive selection plates but no growth on de novo autoactivation plates.

### Split-YFP assay

Protoplasts were isolated from four-week old Col-0 plants according to Yoo et al.^70^. Briefly, leaves were cut into 1 mm wide strips and incubated in an enzyme solution mix [10 mM MES (pH 5.7), 0.4 M Mannitol, 20 mM KCl, 10 mM CaCl_2_, 0.3 g Cellulase R-10 (GoldBio; C8001.0001), 0.1 g Macerozyme R-10 (GoldBio; M8002.0001)] for 4 h at 25 °C. The resulting mixture was filtered and washed in W5 solution. Finally, the protoplasts were suspended in MMG solution [0.4 M Mannitol, 15 mM MgCl_2_, 4 mM MES (pH 5.7) at a final concentration of 3.0 × 10^5^ protoplasts per milliliter.

A split-YFP assay was used to determine PPIs by transforming 1 µg of respective LRR-RK containing plasmid DNA(CD3-1089) and 1 µg of candidate effector containing plasmid DNA (CD3-1096) into freshly isolated protoplast. Transformed protoplasts were incubated at 22 °C for 16 h. The transformed protoplasts were visualized using a Nikon Eclipse 80i microscope.

### Data availability

All supporting data from this study are available from the article and Supplementary Information files, or from the corresponding author upon reasonable request. Moreover, the weighted *k*-shell algorithm implemented in Java language and can be accessed at http://goo.gl/c5ISSe.

## Electronic supplementary material

Supplementary Information

Description of Additional Supplementary Files

Supplementary Data 1

Supplementary Data 2

Supplementary Data 3
