# Supplementary material for: Network biology discovers pathogen contact points in host protein-protein interactomes
Source: Nat Commun. 2018 Jun 13;9:2312. doi: 10.1038/s41467-018-04632-8 (PMC5998135; doi:10.1038/s41467-018-04632-8)
Supplement: Supplementary file 1 — Supplementary Information [file 41467_2018_4632_MOESM1_ESM.pdf]

**Supplementary Information**

# Network biology discovers pathogens contact points in host protein-protein interactomes

Ahmed et al.

## Supplementary Figures

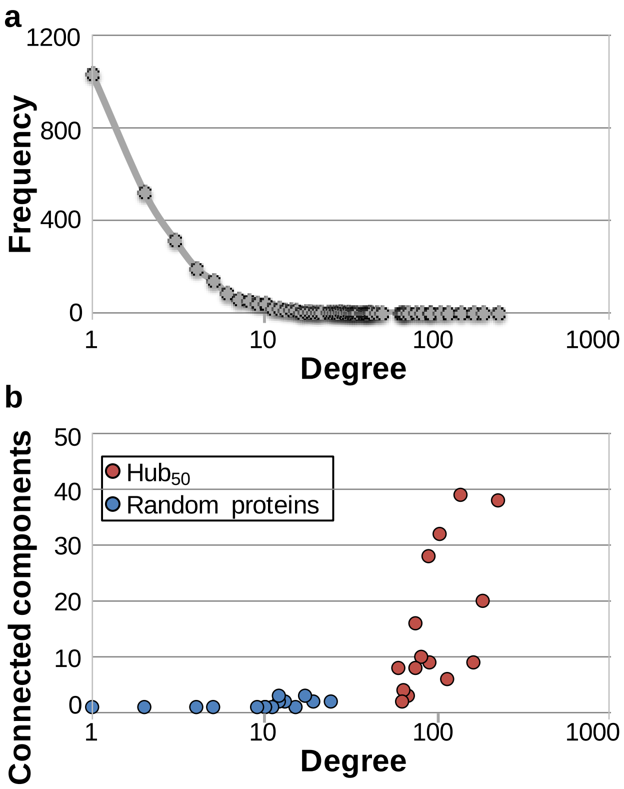


**Supplementary Figure 1: AI-1$_{MAIN}$ displays the properties of scale-free network. a**, AI-1$_{MAIN}$ follows a power-law distribution. The degree of distribution of proteins in AI-1$_{MAIN}$. Degree (log-scale) on x-axis and the frequency on the y-axis are illustrated. Presence of highly connected nodes (hubs) can be observed in the AI-1$_{MAIN}$ network. **b**, High connectivity of AI-1$_{MAIN}$ is demonstrated based on the removal of individual hub (red) or non-hub (blue) in the largest component of AI-1$_{MAIN}$. Elimination of a hub results in formation of significantly higher number of results in the formation of a higher number isolated sub-graphs/nodes compared to the removal of a non-hub.

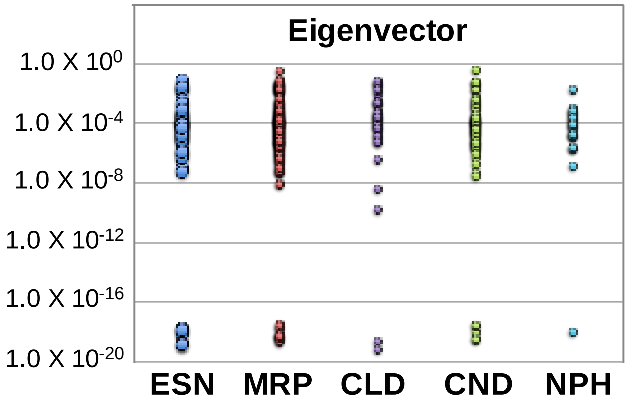


**Supplementary Figure 2: Node distribution corresponding to high eigenvector for five phenotypic groups, essential (ESN), morphological (MRP), conditional (CND), cellular-biochemical (CLB), and no phenotypes (NPH), are displayed.** No association of high eigenvector with two cut-off values with any phenotypic groups was observed.

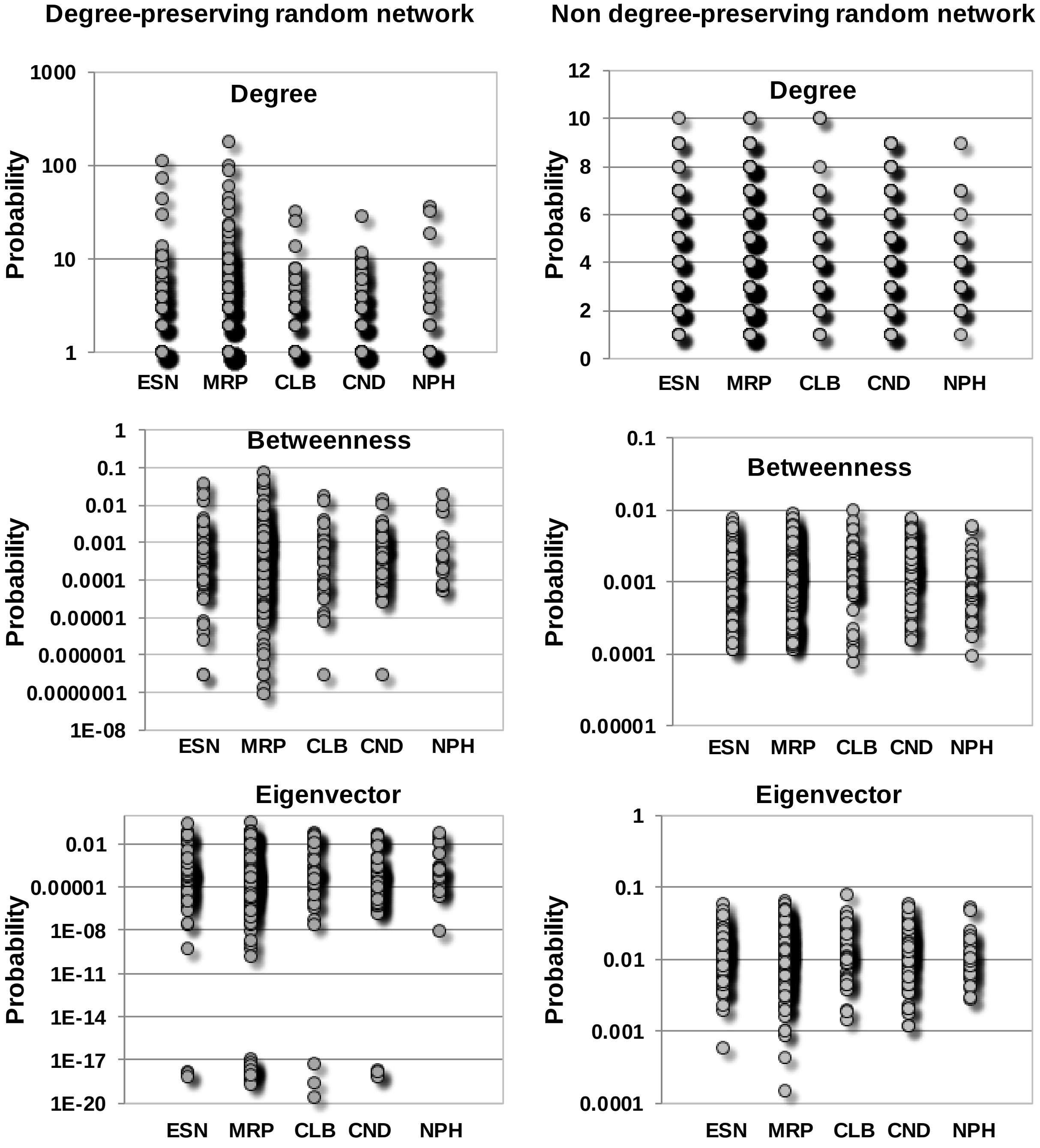


**Supplementary Figure 3: Node distribution corresponding to degree, betweenness, and eigenvector for five phenotypic groups in degree-preserving (left) and non degree preserving networks (right)**. Phenotypes are classified as

essential (ESN), morphological (MRP), cellular-biochemical (CLB), conditional (CND), and no phenotypes (NPH).

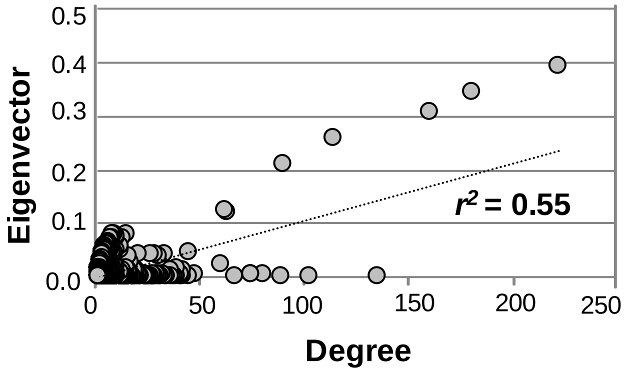


**Supplementary Figure 4: Relationship between degree (x-axis) and eigenvector values (y-axis) in AI-1$_{MAIN}$.** No association of nodes with high degree (hubs) and high eigenvector was observed ($r^2$=0.55).

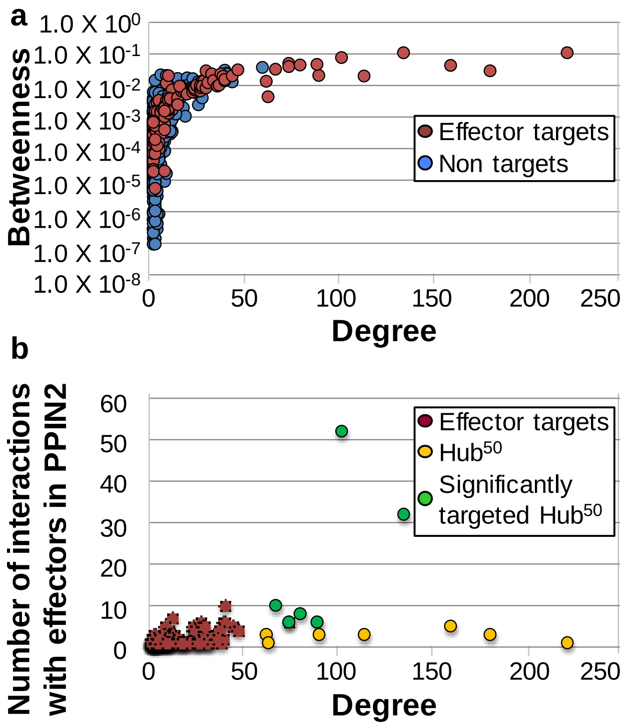


**Supplementary Figure 5: Node attributes as well as node centrality measures and effectors targets. a**, Nodes with high degree/high betweenness (HDHB) are enriched in effector targets. **b**, Correlation between the number of unique effectors interacting with a particular node and its degree in AI-$1_{MAIN}$. Six $hub^{50}$ (green) are interacted significantly more with effectors than expected given their degree in AI-$1_{MAIN}$ ($P < 0.05$). Eight $hub^{50}$ that are not significantly targeted by effectors are illustrated in orange. Non-hub effector targets are displayed in red.

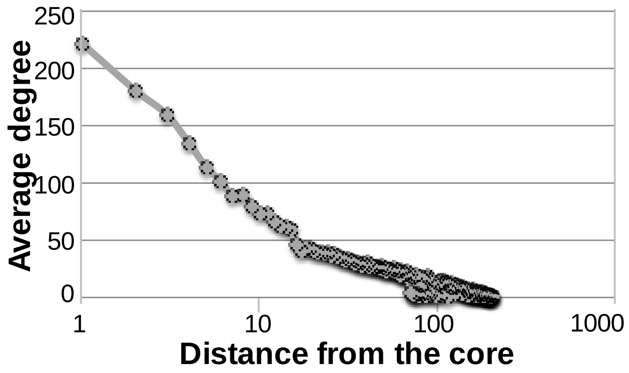


**Supplementary Figure 6: Distribution of average degree of each shell.** Average degree of nodes in each shell is distributed from the innermost of the network (core) designated as 1 to the periphery of the network denoted as 1000 in AI-$1_{MAIN}$ ($r^2$= 0.67 and Mann-Whitney-Wilcoxon Test $P < 2.2 \times 10^{-16}$).

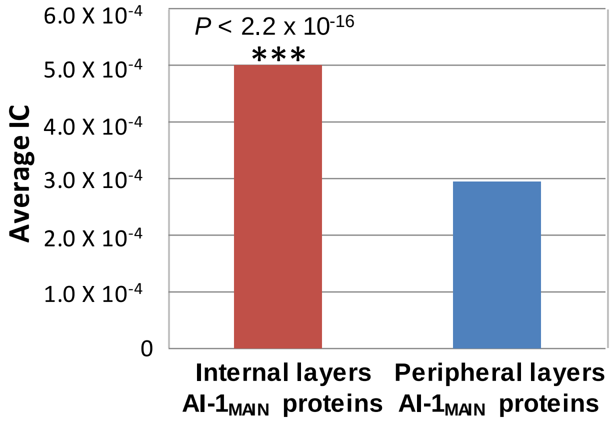


**Supplementary Figure 7: Measurement of average information centrality (IC) of nodes in internal layers AI-$1_{MAIN}$ proteins and peripheral layers AI-$1_{MAIN}$ proteins in Arabidopsis Interactome version 1 (AI-$1_{MAIN}$).** Average IC of internal layers AI-$1_{MAIN}$ proteins is significantly higher than remaining *k*-shell proteins (Welch's t-test $P < 2.2 \times 10^{-16}$).

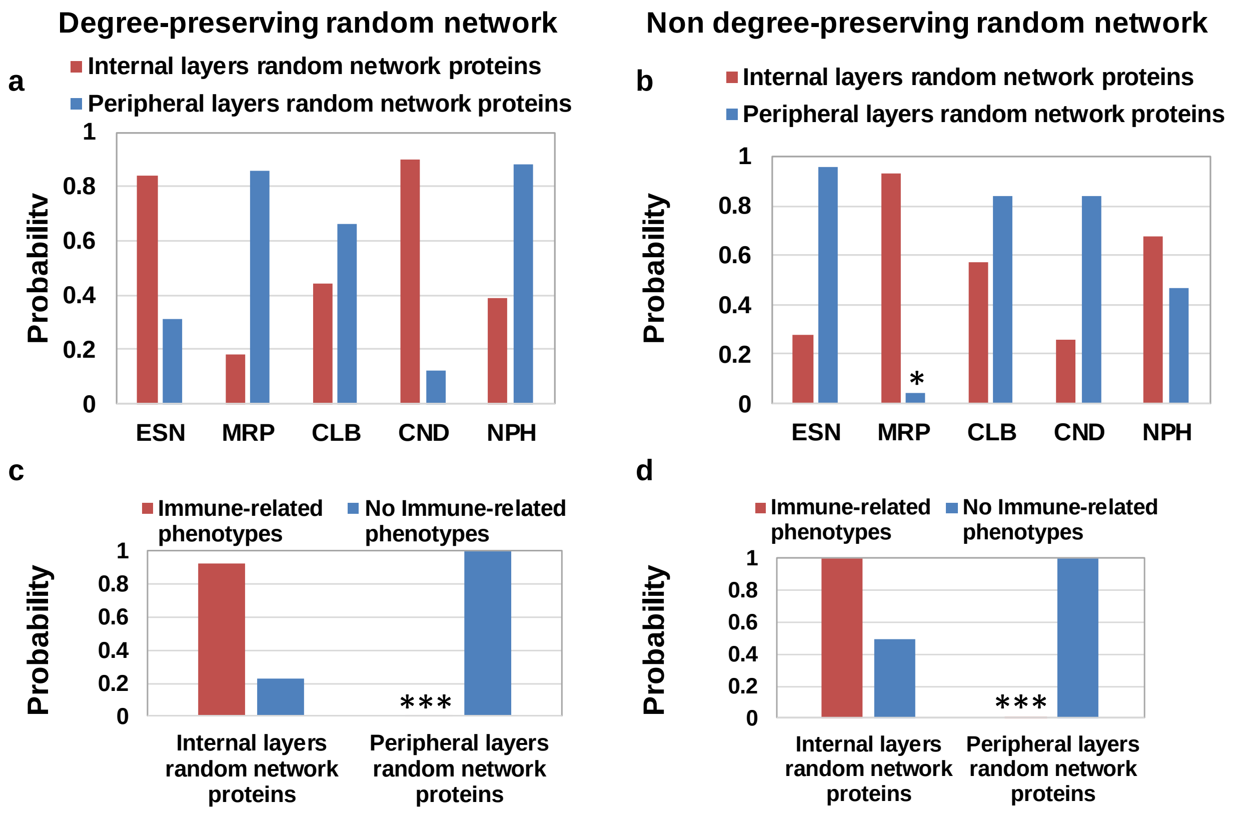


**Supplementary Figure 8: Phenotypic enrichment assays in degree-preserving (a and c) and non degree preserving networks (b and d). a, b,** Phenotypes are classified as essential (ESN), morphological (MRP), cellular-biochemical (CLB), conditional (CND), and no phenotypes (NPH). Nodes for internal and peripheral layers of both random networks are indicated. **c, d,** Enrichment of immune-related (red) and no immune-related phenotypes (blue) for internal and peripheral layers of random networks are shown.

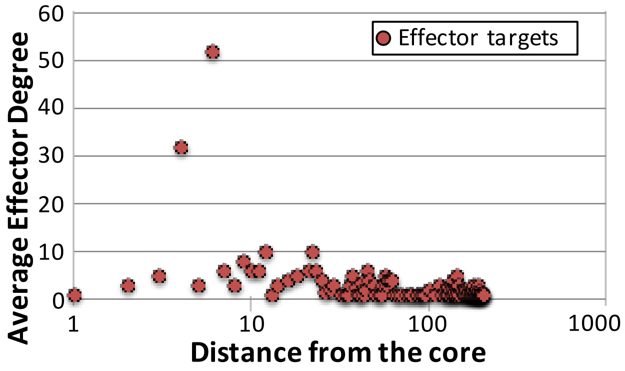


**Supplementary Figure 9: No association of average effector degree and shell location was observed.** AI-$1_{MAIN}$ nodes interacted with number of unique effectors in PPIN-2 are distributed in shells laid out from the core ($k$=1) to the periphery of the network.

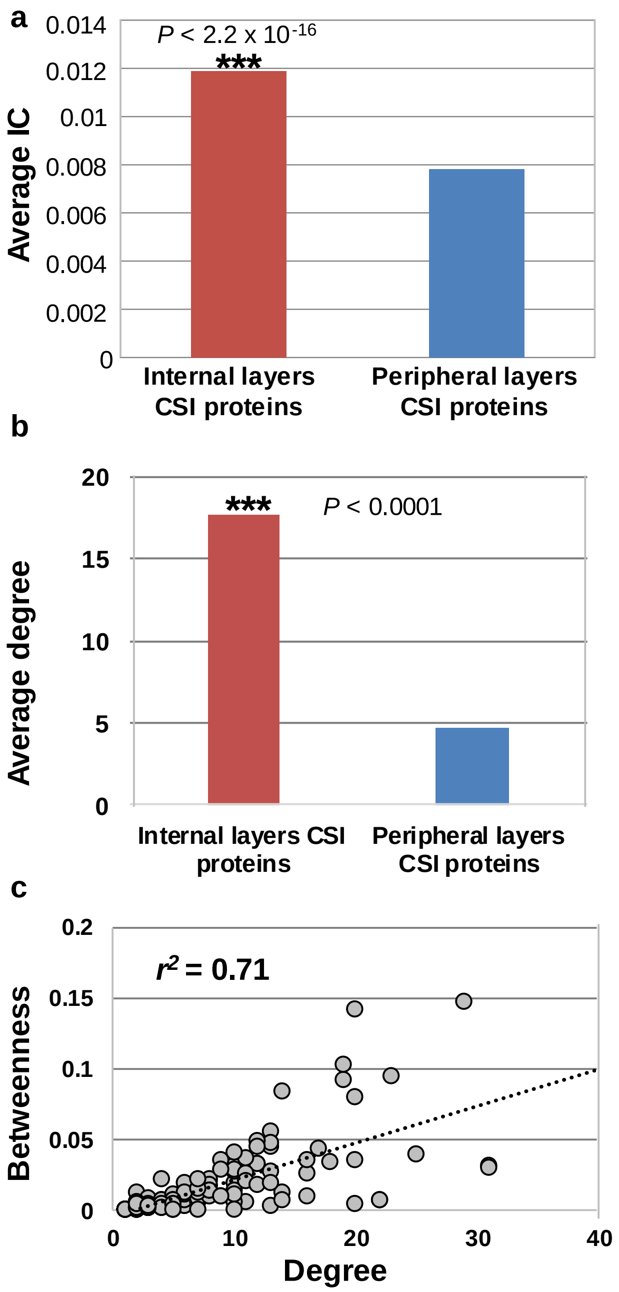


**Supplementary Figure 10: Calculation of average centrality measures in Cell Surface Interactome (CSI$^{LRR}$).** Average information centrality (IC), **a,** average degree, **b** of nodes in internal and peripheral layers proteins of CSI$^{LRR}$ are displayed. **c**,

Relationship between node betweenness and degree distribution**.** *P* and $r^2$ values are indicated.

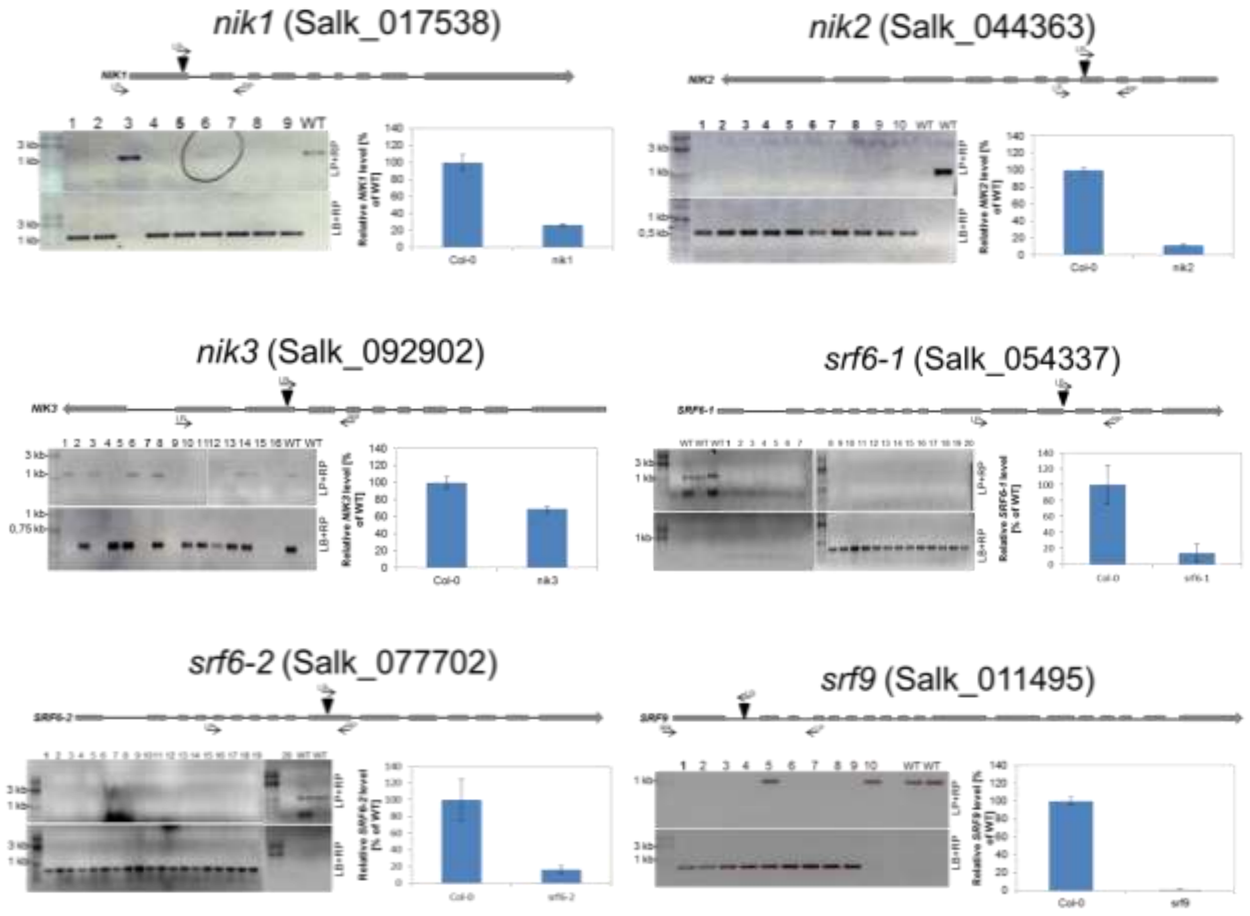


**Supplementary Figure 11: Genotyping of T-DNA lines used in the study.** Each figure details the SALK insertion ID along with a schematic diagram depicting the gene locus and insertion sites. Below gels are showing the LP+RP and BP+RP PCR products for the multiple plants (n>9) tested for each T-DNA line. Appropriate lines used for assays are marked with bold letters. Arrows show binding sites of primers. Bar charts representing the relative expression level in the chosen T-DNA lines as determined by qPCR.
