# Supplementary material for: Network biology discovers pathogen contact points in host protein-protein interactomes
Source: Nat Commun. 2018 Jun 13;9:2312. doi: 10.1038/s41467-018-04632-8 (PMC5998135; doi:10.1038/s41467-018-04632-8)
Supplement: Supplementary file 2 — Description of Additional Supplementary Files [file 41467_2018_4632_MOESM2_ESM.pdf]

Description of Additional Supplementary Files

File Name: Supplementary Data 1
Description: Classification of Arabidopsis genes into five functional groups based on their mutant phenotypes.

File Name: Supplementary Data 2
Description: Hypergeometric analyses to investigate the association of diverse centrality measures with phenotypic groups.

File Name: Supplementary Data 3
Description: Distribution of nodes within internal and peripheral layers in AI-$_1$MAIN and CSI
